# An evaluation of ciprofloxacin pharmacokinetics in critically ill patients undergoing continuous veno-venous haemodiafiltration

**DOI:** 10.1186/1472-6904-11-11

**Published:** 2011-08-04

**Authors:** Almath M Spooner, Catherine Deegan, Deirdre M D'Arcy, Caitriona M Gowing, Maria B Donnelly, Owen I Corrigan

**Affiliations:** 1 School of Pharmacy and Pharmaceutical Sciences, Trinity College Dublin, Dublin 2, Ireland; 2 Intensive Care Medicine, Adelaide and Meath Hospital, Dublin, Incorporating the National Children's Hospital, Tallaght, Dublin 24, Ireland; 3 Pharmacy Department, Adelaide and Meath Hospital, Dublin, Incorporating the National Children's Hospital, Tallaght, Dublin 24, Ireland

## Abstract

**Background:**

The study aimed to investigate the pharmacokinetics of intravenous ciprofloxacin and the adequacy of 400 mg every 12 hours in critically ill Intensive Care Unit (ICU) patients on continuous veno-venous haemodiafiltration (CVVHDF) with particular reference to the effect of achieved flow rates on drug clearance.

**Methods:**

This was an open prospective study conducted in the intensive care unit and research unit of a university teaching hospital. The study population was seven critically ill patients with sepsis requiring CVVHDF.

Blood and ultrafiltrate samples were collected and assayed for ciprofloxacin by High Performance Liquid Chromatography (HPLC) to calculate the model independent pharmacokinetic parameters; total body clearance (TBC), half-life (t_1/2_) and volume of distribution (Vd). CVVHDF was performed at prescribed dialysate rates of 1 or 2 L/hr and ultrafiltration rate of 2 L/hr. The blood flow rate was 200 ml/min, achieved using a Gambro blood pump and Hospal AN69HF haemofilter.

**Results:**

Seventeen profiles were obtained. CVVHDF resulted in a median ciprofloxacin t_1/2 _of 13.8 (range 5.15-39.4) hr, median TBC of 9.90 (range 3.10-13.2) L/hr, a median V_dss _of 125 (range 79.5-554) L, a CVVHDF clearance of 2.47+/-0.29 L/hr and a clearance of creatinine (Cl_cr_) of 2.66+/-0.25 L/hr. Thus CVVHDF, at an average flow rate of ~3.5 L/hr, was responsible for removing 26% of ciprofloxacin cleared. At the dose rate of 400 mg every 12 hr, the median estimated C_pmax_/MIC and AUC_0-24_/MIC ratios were 10.3 and 161 respectively (for a MIC of 0.5 mg/L) and exceed the proposed criteria of >10 for C_pmax_/MIC and > 100 for AUC_0-24_/MIC. There was a suggestion towards increased ciprofloxacin clearance by CVVHDF with increasing effluent flow rate.

**Conclusions:**

Given the growing microbial resistance to ciprofloxacin our results suggest that a dose rate of 400 mg every 12 hr, may be necessary to achieve the desired pharmacokinetic - pharmacodynamic (PK-PD) goals in patients on CVVHDF, however an extended interval may be required if there is concomitant hepatic impairment. A correlation between ciprofloxacin clearance due to CVVHDF and creatinine clearance by the filter was observed (r^2 ^= 0.76), providing a useful clinical surrogate marker for ciprofloxacin clearance within the range studied.

**Trial Registration:**

Current Controlled Trials ISRCTN52722850

## Background

Severe sepsis is a significant contributor to Intensive Care Unit (ICU) admission and reports vary from 12% to 27% of ICU admissions in different countries [1]. Many more patients develop sepsis following ICU admission. EPIC 2 demonstrated that 51% of ICU inpatients were classified as infected on the day of the point prevalence study. In this study 62% of isolates were identified to be Gram negative, which is worrisome given the dearth of in-development antimicrobials with gram negative coverage [2]. Under dosing of antibiotics has enabled the genesis of resistant strains and this is particularly an issue with fluoroquinolones, aminoglycosides and beta lactams [3,4]. Of particular concern is the ability of fluoroquinolones to engender resistance to other classes of antibiotics [5]. Altered drug pharmacokinetics, due to disease, results in variable antimicrobial drug clearance in critically ill patients (antibiotic regimens are often developed on the basis of drug disposition in non-critically ill volunteers) and further complicates the selection of appropriate dosing schedules for these patients. For optimal dosing, it is necessary to consider the kill characteristics of the antibiotic.

Pharmacokinetic-pharmacodynamic (PK-PD) parameters can be used to indicate the potential for bacterial eradication with antimicrobial therapy. Bactericidal activity can be time-dependent or concentration-dependent. Quinolones exhibit concentration-dependent bacterial killing [6-10]. Consequently bactericidal activity becomes more pronounced as serum drug concentrations increase to approximately 10 times the minimum inhibitory concentration (MIC.). The goal of ciprofloxacin therapy is to maximise the 24-hr AUC/MIC and the peak/MIC ratios [10]. There is general concern about the emergence of resistance as a result of inadequate doses of ciprofloxacin [9]. A C_pmax_/MIC ratio, where C_pmax _is the maximum steady state serum concentration, of >10 and an AUC_24_/MIC ~> 100 have been proposed as predictors of therapeutic efficacy [10-12]. In clinical practice, C_pmax _is equated with the serum peak level. A number of papers have highlighted the requirement for a re-evaluation of currently recommended antimicrobial dosage regimens for critically ill patients [13,14].

Ciprofloxacin disposition is affected by critical illness, particularly by the presence of organ failure and dosage adjustment is advised in patients with renal failure [15]. In patients with severely impaired renal function, a 50% dosage reduction has been recommended [16]. Some data are extrapolated from non-critical patients with renal failure and others from critically ill patients without renal failure. Doses ranging from 200 mg twice daily to 400 mg three times daily have been used for critically ill patients without renal impairment [17,18].

Renal elimination, including both glomerular filtration and tubular secretion accounts for approximately 66% of ciprofloxacin clearance [19]. In healthy volunteers, the hepatic route accounts for approximately 20% of elimination, with transintestinal excretion also playing a possibly significant role. This is thought to represent a notable compensatory pathway preventing drug accumulation in renal failure. In terms of clearance during continuous renal replacement therapy (CRRT), the method of CRRT used has been presented as an important determinant of the effect of CRRT on clearance, with increased drug clearance via CVVHDF compared to continuous veno-venous haemofiltration (CVVH) [20]. Nonetheless, clearance via CVVH of up to 25% has been reported [21]. It has been recommended therefore that dosing during CVVHDF is focussed on attaining clinically adequate drug concentrations, preferably with concurrent therapeutic drug monitoring.

As a result of the reported variability in ciprofloxacin pharmacokinetic parameters during critical illness, differences in patient populations and CRRT conditions in literature reports and the absence of a consensus on dosing regimens, a prospective pharmacokinetic evaluation of ciprofloxacin during CVVHDF therapy was undertaken.

## Methods-

### Patient Demographics and Clinical Characteristics

This was an open, prospective pharmacokinetic study in a multidisciplinary, intensive care unit in a university teaching hospital. Ethics approval was obtained from the Joint Ethics Committee (St James's/AMNCH) (Reference Number 041008/7804). Clinical trial approval was granted by the Irish Medicines Boards (EudraCT Number 2004-002195-42) and the trial was registered with Current Clinical Trials (ISRCTN52722850). Consent (predominantly consent by proxy) was obtained in compliance with Helsinki declaration. Seven critically ill patients, treated concurrently with intravenous ciprofloxacin and CVVHDF therapy, were enrolled in the study. Intravenous ciprofloxacin 400 mg twice daily administered as a one hour infusion was the dosage regimen generally employed, dosing at all times was at the discretion of the physician. A dosage regimen of ciprofloxacin 400 mg once daily was also analysed for three patients, while a dosage regimen of 200 mg twice daily was also assessed in one patient. MIC susceptibility testing for pathogens isolated was not performed. Instead a representative MIC of 0.5 mg/L was employed based on an analysis of local ecology data. It should be noted that CVVHDF patients in this hospital are prescribed on average 14.3 drugs during CVVHDF, 4.7+/-2.66 are anti-infectives.

### CVVHDF conditions

CVVHDF was performed at prescribed dialysate rates of 1 or 2 L/hr and ultrafiltration rate of 2 L/hr. This reflects the typical CVVHDF prescription of the unit. The blood flow rate was 200 ml/min, achieved using a Gambro blood pump and Hospal AN69HF haemofilter. For patients 6 and 7, CVVHDF was run heparin-free, due to coagulopathy.

### Measurement of Ciprofloxacin Concentrations

Timed serum samples were collected during each dosage interval and ultrafiltrate during 7 dosage intervals (1 per patient). Effluent fluid was collected for the entire dosage interval. The volume of each hourly batch was recorded and a 40 ml sample was taken for analysis. Aliquots from each sample were analysed for ciprofloxacin concentration and for creatinine determination. Total ciprofloxacin concentrations in serum and effluent were measured by the HPLC method of Davis et al [22], adapted for both serum and effluent fluid analysis. Quantitation was based on external standard calibration using the ratio of the peak areas of the analyte and the internal standard (β-hydroxypropyl theophylline). Replicate analysis was performed both on control samples and study samples. Ciprofloxacin hydrochloride monohydrate (1g) (gift from Bayer UK) was used to verify the concentration of the commercial infusion solution, Ciproxin^®^. The extraction efficiency was in excess of 80% in the concentration range 0.5-20.0 μg/ml and the between day coefficient of variation <10%. Precision was less than 5.0 R.S.D.%. The sensitivity of the assay was 0.5 μg/ml.

### Analysis of Serum Concentrations of Ciprofloxacin

Serum concentrations, from an indwelling arterial cannula, were measured immediately before the infusion was started, immediately after the infusion finished and at 2,3,4,6,8 and 12 hours post infusion where the dosage interval was 12 hr. When the prescribed dosage interval was 24 hr samples were also taken at 18 and 24 hrs. Exact sampling times were recorded. Thus C_pmax _was directly measured.

### Pharmacokinetic analysis

#### Calculating half-life and clearance

Non-compartmental pharmacokinetic methods were used. Pharmacokinetic analysis was performed using WinNonlin pharmacokinetic software, version 5.2, (Pharsight Corporation, North Carolina, U.S.A.). The terminal half-life (t_1/2_) was calculated as 0.693/λ_z_, where λ_z _is the first order terminal elimination rate constant. The area under the plasma concentration-time curves (AUC) were calculated using the linear trapezoidal method. AUC for the study period (n = 12 or 24 hours) was used to calculate the AUC extrapolated to infinity (AUC_0-∞_) by the equation AUC_0-t* _+ C*/λ_z_. where C* is the final plasma concentration, at the final sampling time, t*. The Total Body Clearance (TBC) was calculated as dose/AUC_0-∞ _for profile 4a as this was a first dose, and as dose/AUC_0-τ _at steady state, where τ is the dosage interval, for all other profiles except 2c, 4b and 6c, which were not at steady state.

#### Estimating clearance for profiles not at steady state

Profiles 2c, 4b and 6c did not result from initial doses, and could not be considered to be at steady state as they directly followed a change in dose or interval. For these profiles, TBC was estimated as dose/AUC_n0-∞_.

AUC _n0-∞ _in these cases was estimated as (AUC_0-∞ _-(C*_(n-1)_/λ_z __(n-1)_)), where C*_(n-1) _was the final observed concentration at the end of the preceding dose interval, and λ_z __(n-1) _was the first order terminal elimination rate constant calculated from the preceding dose interval.

#### Calculating volume of distribution at steady state (V_dss_)

Because of the severity of illness body weights could not be accurately monitored, consequently parameters were not weight normalised. Volume of distribution at steady state (V_dss_) for an initial dose was calculated as , where t_inf _is the duration of the infusion, and as  from profiles at steady state. In cases where the dosing interval was changed from 24 (at steady state) to 12 hours, the first profile following the change was calculated as a 24 hour interval, with sampling ceasing at 12 hours. This was the case for profiles 6b and 7b. V_dss _was not calculated for profiles 2c, 4b and 6c as these were neither an initial dose or at steady state.

#### Calculating sieving coefficients

Sieving coefficients (S) for ciprofloxacin and creatinine were calculated from the time-matched concentrations in effluent and in serum for a single dosage interval, whereby S_creatinine _= C_effluent_/C_serum _and S_cipro _= C_effluent_/C_serum_. The clearance of creatinine was calculated from S_creatinine _and the measured flow of effluent (Q) where Cl_creat _= S_creat _x Q.

The fraction cleared by CVVHDF (F_CVVHDF_) was determined from Cl_CVVHDF_/TBC. The PK-PD parameters C_pmax_/MIC and AUC_0-24 _/MIC were employed as predictors of the likelihood of clinical and microbiological response.

## Results

### Patients

Relevant demographic and clinical data relating to the seven patients studied are presented in Table 1. Patients were severely ill having renal failure, haemodynamic instability and coagulopathy. Six patients were prescribed ciprofloxacin for documented infection *(Pseudomonas aeruginosa, Escherichia coli)*, while one patient with suspected sepsis was prescribed ciprofloxacin empirically. The median APACHE Π score was 27 (range 25-30).

**Table 1:** Demographic and clinical data of patients on continuous veno-venous haemodiafiltration administered ciprofloxacin

	Sex	Age	Diagnosis	Infective pathogen	APACHE II score(initial/highest)	CVVHDF Duration(days)	ICU Mortality Outcome
1	M	60	Intestinal Obstruction, Hemicolectomy	*Escherichia coli*	26	6	Survived

2	F	77	Neutropaenic sepsis	*Pseudomonas aeruginosa*	27	5	Died

3	F	68	Intestinal obstruction, post-operative sepsis and acute renal failure	*Pseudomonas aeruginos*a	28	14	Survived

4	M	47	Acute pancreatitis, sepsis	*Escherichia Coli*	25	14	Survived

5	F	71	ESRD with severe sepsis	Empiric cover	27	8	Died

6	M	57	Hepatic cirrhosis with severe sepsis	*Pseudomonas Aeruginosa, Enterococcus faecalis*	29	7	Survived

7	M	28	Acute liver failure with severe sepsis	*Pseudomonas aeruginosa*	30	11	Survived

### CVVHDF conditions

The actual flow rates achieved were recorded and compared with the target flow rates. The mean effluent flow rate achieved was 3.35 +/- 0.50 L/hr (Range: 2.9 - 4.0 L/hr). The mean duration of CVVHDF therapy was 9.3 +/- 3.7 days. The mean number of filters used per dosage interval was 1.1. The mean duration of use of a filter was 50.6 hours.

### Pharmacokinetic profiles

Seventeen pharmacokinetic profiles were obtained from these seven patients. The ciprofloxacin pharmacokinetic parameters estimated from each patient's pharmacokinetic profile, during treatment with CVVHDF are presented in Table 2. Ciprofloxacin t_1/2 _during CVVHDF was variable, ranging from 5.15 to 39.4 hours, with a median of 13.8 hours, reflecting a median elimination rate constant of 0.050 (range 0.018-0.135) hr^-1^. The average t_1/2 _of patients 1 and 4 (both had hepatic impairment) were 37.3 and 25.9 hours, respectively. These t_1/2 _values are seven to eight times that obtained in patients with normal renal function, and were the highest obtained in the current study. Patient 4 had evidence of liver injury that may have resulted in impaired biliary clearance, and patient 1 had concurrent alcoholic liver disease, which may have contributed to the prolonged t_1/2_. In patient 4, profile 4a, the clearance was calculated using the estimated AUC_0-∞_, as this profile was measured following the initial dose. The long half life calculated (25 h) in combination with the lower volume of distribution lead to a comparatively low estimated TBC. The t_1/2 _observed in patients 3 and 5, 6.73 and 7.00 respectively, were closer to those seen in patients with normal renal function. The median TBC of ciprofloxacin was 9.90 (range 3.10-13.2) L/hr (~0.14 L/hr/kg based on a 70 kg patient). This value represents hepatic, residual renal and transintestinal ciprofloxacin clearance, in addition to ciprofloxacin clearance by the filter. The V_dss _for ciprofloxacin during CVVHDF therapy ranged from 79.5-555 L with a median of V_dss _of 125 L. The mean C_pmax _concentration following administration of 400 mg every 12 hours was 5.8 +/- 1.0 mg/L.

**Table 2:** Estimates of pharmacokinetic parameters obtained from multiple ciprofloxacin serum concentrations in a dosage interval using non-compartmental methods

Patient Profile	Dose (mg)	Dosage Interval (hours)	T_1/2 _(hrs)	k (hr^-1^)	AUC_0-τ_* _(τ = dosage_ _interval)_ (mg.hr/L)	TBC (Dose/AUC) (L/hr)	Vd_ss_ (L)
1.A	400	12	35.2	0.020	35.4	11.3	524
1.B	400	12	39.4	0.018	38.3	10.4	555
**Mean**			**37.3**	**0.019**	**36.8**	**10.9**	**539**

2.A	200	12	5.15	0.135	15.2	13.2	104
2.B	200	12	13.8	0.050	16.5	12.2	215
2.C	400	12	12.4	0.056	51.8	7.72	
**Mean**			**10.5**	**0.080**		**11.0**	**160**

3.A	400	12	5.97	0.116	38.9	10.3	79.5
3.B	400	12	7.50	0.092	40.2	10.0	97.3
**Mean**			**6.73**	**0.104**	**39.5**	**10.1**	**88.4**

4.A	400	24	24.7	0.028	129	3.10	111
4.B	400	12	27.2	0.026	81.5	4.91	
**Mean**			**25.9**	**0.027**		**4.00**	

5.A	400	12	8.44	0.082	40.4	9.90	107
5.B	400	12	5.77	0.120	37.8	10.6	81.1
5.C	400	12	6.80	0.102	40.9	9.78	88.2
**Mean**			**7.00**	**0.101**	**39.7**	**10.1**	**92.2**

6.A	400	24	14.0	0.050	43.4	9.22	175
6.B	400	12	14.7	0.047	54.0	7.40	151
6.C	400	12	16.5	0.042	49.6	8.06	
**Mean**			**15.0**	**0.046**		**8.23**	**163**

7.A	400	24	15.1	0.046	50.6	7.91	164
7.B	400	12	10.4	0.067	39.4	10.1	139
**Mean**			**12.7**	**0.056**		**9.00**	**151**

* τ taken to be 24 hours for first profile immediately following a change in prescribed dosage interval (profiles 6b and 7b)

The clearances of ciprofloxacin and creatinine by CVVHDF are presented in Table 3. The mean clearance of ciprofloxacin by CVVHDF was 2.47 +/- 0.29 L/hr, thus the clearance of ciprofloxacin by CVVHDF was on average 26% of the TBC, reflected in the mean F_CVVHDF _of 0.26 (Table 3). This value excludes that of patient 4. In this case a significant fraction (0.74) of the clearance is calculated to have occurred via CVVHDF, and was therefore considered an atypical value.

**Table 3:** Ciprofloxacin and Creatinine Clearance by continuous veno-venous haemodiafiltration.

Patient	Cl_CVVHDF_ (L/hr)	Cl_CREAT_ (L/hr)	F_CVVHDF_	Measured effluent fluid rate (L/hr)	Total Body Clearance (L/hr)
1 A	2.8	2.9	0.25	4.0	11.3
2 C	2.4	2.6	0.31	3.4	7.72
3 B	2.7	2.9	0.27	3.9	9.96
4 A	2.3	2.3	0.74	2.9	3.10
5 A	2.2	2.6	0.22	3.0	9.90
6 C	2.1	2.4	0.26	2.9	8.06
7 B	2.8	2.9	0.28	4.0	10.1

**Mean +/- SD**	**2.47 +/- 0.29**	**2.66 +/- 0.25**	0.26 ± 0.03*	**3.35 +/- 0.50**	**8.60 ± 2.7**

* Excluding value from patient 4A

**Figure 1:**
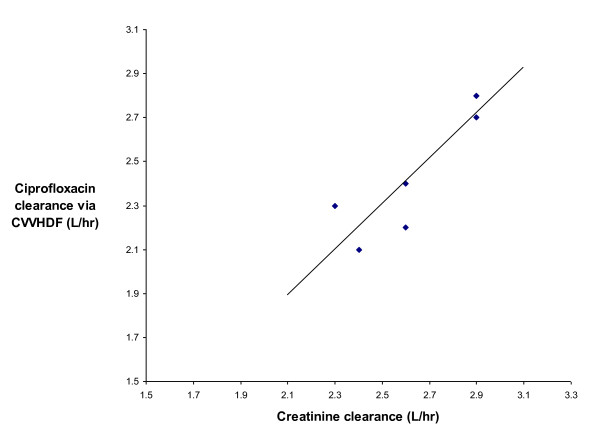
**Relationship between ciprofloxacin clearance via CVVHDF (L hr^-1^) and creatinine clearance (L hr^-1^)**.

The S_cipro _was 0.70 +/- 0.06. A simple method for estimating drug clearance through the filter, without measuring drug levels, involves using the non-protein bound fraction (f_u_) of ciprofloxacin as an estimate of the sieving coefficient, as it is the unbound fraction that crosses the filter. Hoffken et al [23] and Joos et al [24] have reported f_u _values for ciprofloxacin of 0.6 and 0.78 respectively. Applying these values to the observed flow rates in this study gives clearance estimates of 2.0 L/hr and 2.7 L/hr, which approximate the measured value of 2.47 L/hr. The sieving coefficient for creatinine (0.82 +/- 0.04) was quite similar to that estimated for ciprofloxacin. A correlation between ciprofloxacin clearance due to CVVHDF (y) and creatinine clearance by the filter (x) was observed (y = -0.29 + 1.03x, r^2 ^= 0.76) and is illustrated in Figure 1. Ciprofloxacin and creatinine clearances over time were examined in order to identify any change in filter efficiency over time. There was little variation in filter performance as the clearance of both creatinine and ciprofloxacin remaining relatively constant.

**Figure 2:**
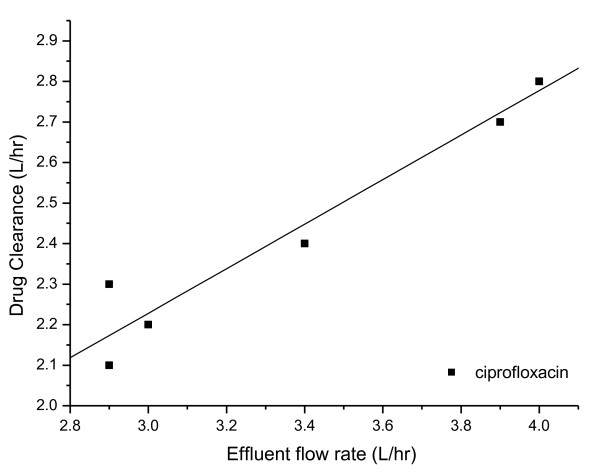
**Relationship between ciprofloxacin clearance by CVVHDF (L hr^-1^) and effluent flow rate (L hr^-1^)**.

Within the observed range of effluent flow rates and CVVHDF ciprofloxacin clearance rates in the current study, there is a suggested trend (r^2 ^= 0.94) of increasing ciprofloxacin clearance with higher effluent fluid flow rates, which is illustrated in Figure 2.

### Pharmacokinetic - Pharmacodynamic parameters

The PK-PD parameters, C_pmax_/MIC ratios and the ratio of AUC_0-24 _/MIC, achieved with a ciprofloxacin dosing regimen of 400 mg ciprofloxacin every 12 hours are summarised in Table 4.

**Table 4:** Cpmax/MIC ratios for representative MICs for patients treated with 400 mg ciprofloxacin every 12 hours during critical illness and continuous veno-venous haemodiafiltration therapy.

Patient ID	Cpmax/MIC ratio (MIC = O.5 mg/L)	AUC_0-24_/MIC ratio (MIC = O.5 mg/L)
1	10.3	147
2	10.2	207
3	12.8	158
5	14.6	159
6	10.0	199

The median AUC_0-24_/MIC ratio for patients administered Ciprofloxacin 400 mg twice daily was 161. An AUC_0-24_/MIC~ > 100 has been propounded as an indicator of adequate ciprofloxacin dosing [12].

## Discussion

The pharmacokinetic parameter estimates obtained, half life [median 13.8 (range 5.15-39.4) hours], TBC [median 9.90 (range 3.10-13.2) L/hr] and median V_dss _of 125 (range 79.5-555) L illustrate the high level of inter-patient variability in ciprofloxacin disposition in critically ill patients during CVVHDF. The t_1/2 _of ciprofloxacin, approximately 4 hours in patients with normal renal function, doubles in patients with severe renal impairment. In general, accumulation will not be observed with 12 hour dosing as this interval is greater than the half-life, in patients without liver dysfunction. As significant accumulation was observed with an interval of eight hours in previous studies [15,17,25] a longer dosage interval of 12 hours for patients on CVVHDF should therefore be considered. Patients 1,4,6 and 7 had average half-lives varying from 13-15 hours (patients 6 and 7) to greater than 25 hours (patients 1 and 4), demonstrating the variability of pharmacokinetic profiles in this patient group, and also likely reflecting decreased hepatic clearance in these four patients.

Wallis et al [15] reported a similarly reduced ciprofloxacin clearance in six patients with renal failure, treated with 200 mg ciprofloxacin three times daily during CVVHDF therapy (0.06-0.25 L/hr/kg).

The median volume of distribution of 125 L (mean 185 +/- 155) is similar to values reported by Wallis et al [15] (mean: 135+/- 27 L) estimated from six patients treated with 200 mg every 8 hours and by Lipman et al [18] (range: 0.77 - 2.52 L/kg) for critically ill patients. The current patient sample showed greater variability: Patient 1, in particular, had a very high V_dss _of 539L. The average V_dss _for the other 6 patients was 126+/-43L. These values compare well with those detailed above from Wallis et al [15], and probably reflect more typical V_dss _values of ciprofloxacin in this patient population. However, the high V_dss _calculated from patient 1 serves as evidence of the unpredictable drug disposition which can occur in critically ill patients, reflecting greater degrees of capillary leak in keeping with the severity of illness.

A practical and useful finding was that, within the range studied, creatinine clearance may serve as a clinical surrogate for ciprofloxacin clearance on CVVHDF. This relationship is clinically important as ciprofloxacin is not routinely assayed in most hospitals. The relationship did not decline over the life of the filter. This relationship may serve as a useful guide to dosing ciprofloxacin within this range of creatinine clearance values.

As effluent flow rates increased, there was a suggested trend of increased ciprofloxacin clearance via CVVHDF. However, there was no observed change in TBC with increasing effluent flow rate. This illustrates the varying role of compensatory and alternative elimination methods such as hepatic elimination. Further studies on this association between effluent flow rate and ciprofloxacin clearance via CVVHDF to investigate the presence of a cause-effect relationship would be beneficial and may helpfully influence dosing decisions. This is of importance in particular as increased effluent flow rates may be used for purposes other than enhancing drug clearance, and therefore TBC in such patients can be difficult to estimate in the absence of plasma concentration data and due to the varying influence of alternative elimination routes. Further insight into the effect of increasing effluent flow rate on clearance via CVVHDF is therefore desirable. Prospectively, such dosing considerations are often hampered by the discrepancy between prescribed and achieved flow rates.

CVVHDF was responsible for clearing approximately one quarter of all ciprofloxacin eliminated. Its relative contribution to ciprofloxacin TBC will be greatest in patients with concurrent hepatic and renal dysfunction, as in these patients, CVVHDF will become a proportionately more significant route. Therefore care is required in dosing patients with concurrent renal and hepatic failure/impairment to avoid accumulation of ciprofloxacin. Interestingly, clearance of ciprofloxacin via CVVH of up to 25% has been reported [21], which is similar to that observed in the current study using CVVHDF. This implies that both dialysis parameters and patient specific parameters have a role to play in the observed variability in clearance via CRRT, in addition to the CRRT method used.

There is now significant evidence that correct and timely antibiotic choices will save more lives than virtually all other ICU therapies [4,26]. A C_pmax_/MIC ratio of ~>10 has been suggested [9,11] as desirable and thus a 400 mg twice daily regimen appears to achieve these target concentrations during CVVHDF therapy (Table 4), 200 mg twice daily being inadequate. For ciprofloxacin 400 mg twice daily, the median C_pmax _achieved in this study was 5.2 (range 5.0-7.3) mg/L (mean 5.8 ± 1.0 mg/L), which represented a median C_pmax_/MIC ratio (based on an MIC of 0.5 mg/L) of 10.3. The median AUC_0-24 _was 80.4 (range 70.8-104) mg.hr/L (mean 83.0 ± 11.1 mg.hr/L), which corresponds to a median AUC_0-24_/MIC ratio of 161 (range 142-207). A suggested characteristic of adequate dosing for ciprofloxacin is an AUC_0-24_/MIC ratio > 100.

Wallis et al [15] reported lower C_pmax _concentrations and AUC_0-24 _values with a lower daily dose of 600 mg ciprofloxacin, compared to the 800 mg daily dose used in this study. The CVVHDF conditions in our study were similar to those reported by Wallis et al [15]. Wallis et al [15] used a dosing schedule of 200 mg every 8 hours and the mean C_pmax _concentration was 3.5 +/- 0.5 mg/L. This dosing schedule achieves on average a C_pmax_/MIC ratio of 7 (based on a MIC of 0.5 mg/L). The mean AUC_0-24 _achieved by the same dosing schedule of 200 mg every 8 hours was 48.3 +/- 8.7 mg.h/L, equivalent to a AUC_0-24_/MIC ratio of 96.6 +/- 17.4, on the basis of a MIC value of 0.5 mg/L. Malone et al [27] reported a mean C_pmax _of 3.9 mg/L (C_pmax _/MIC ratio; 7.8) for three patients treated with 400 mg ciprofloxacin every 24 hours during CVVHDF therapy. The mean AUC_0-24 _with this dosing schedule was 56.8 mg.h/L, which represents an AUC_0-24_/MIC ratio of 114 (assuming an MIC of 0.5 mg/L). The CVVHDF conditions employed by Malone et al [27] were different from those used in the current work.

On the basis of these pharmacodynamic considerations, the dosing schedule utilised in this study gives better cover than previously studied dosage strategies and should serve as a pharmacokinetically and pharmacodynamically valid dose guide in the appropriate clinical circumstances.

One limitation of this work is common to all carried out in this field, that patient numbers are small, and as such the conclusions that can be drawn are limited to a very narrow patient cohort. A consideration for future research might be that a randomised crossover design could be employed to add further clarity to dosing recommendations. As both antimicrobial doses, and dialysis parameters such as flow rate, are frequently adjusted on the basis of changing clinical need in the intensive care setting, there are ethical difficulties surrounding the set up and design of such trials.

Another limitation was that liver dysfunction or impairment was not characterised in a standardised manner. However this is difficult in these patients due to blood product and vitamin K administration, particularly in septic and coagulopathic patients and also because of concurrent hepatotoxic drug administration. Similarly, the varying nature from day to day of hepatic/renal function in many critically ill patients suggest that calculations based on steady state be interpreted with caution, as clearance is likely to be variable. Furthermore, it is extremely difficult to quantify any residual renal function, and thereby contribution to the clearance, that these patients made have had.

## Conclusion

Our results suggest that a dose rate of 400 mg every 12 hours, will achieve the recommended PK-PD goals in *most *patients on CVVHDF. Twice daily dosing of ciprofloxacin maximised peak concentrations, while minimizing accumulation. Concomitant hepatic and renal dysfunction results in a prolonged elimination half-life. Creatinine clearance by the filter may be used to estimate ciprofloxacin clearance by CVVHDF (within the range studied). Thus in the absence of ciprofloxacin plasma levels, creatinine clearance by the filter could be considered as a surrogate marker for ciprofloxacin clearance in patients, this may be particularly useful when other routes of elimination are impaired.

The data suggest that drug clearance increases with increasing effluent flow rate, which in turn may require even higher ciprofloxacin dosing. This association between effluent flow rate and drug clearance requires further elucidation to determine the nature of any cause-effect relationship.

While a 400 mg dose administered at 12 hourly intervals achieved adequate C_pmax _concentrations and lower doses may result in target serum concentrations in some patients with hepatic dysfunction, given the general emergence of ciprofloxacin resistance and the wide therapeutic index of ciprofloxacin, it may be advisable to maintain the higher ciprofloxacin dose as the mainstay of therapy. The possibility of accumulation in patients where more than one elimination route is impaired should be considered and this can be addressed by extension of the dosage interval.

## Key Messages

• Ciprofloxacin 400 mg twice daily IV will achieve the recommended PK-PD goal (AUC_0-24_/MIC ratio >125) in most patients on CVVHDF.

• Concomitant hepatic and renal impairment results in a prolonged ciprofloxacin elimination half life, therefore dosage interval extension may be required.

• Creatinine clearance by the filter correlates well with ciprofloxacin clearance by the filter, therefore creatinine clearance by the filter (in the range studied) may be used as a surrogate for ciprofloxacin clearance.

## List of abbreviations

APACHE II: Acute Physiology and Chronic Health Evaluation score; AUC: Area Under the Curve; AUMC: Area Under the Moment Curve; C*: Final plasma concentration; Cl_cr: _Clearance of creatinine; C_pmax: _Maximum plasma concentration; CRRT: Continuous Renal Replacement Therapy; CVVHDF: Continuous Veno-Venous Haemodiafiltration; ESRD: End Stage Renal Disease; F_CVVHDF: _Fraction cleared by continuous veno-venous haemodiafiltration; f_u: _Non protein bound fraction; HPLC: High Performance Liquid Chromatography; ICU: Intensive Care Unit; LFTs: Liver Function Tests; MIC: Minimum Inhibitory Concentration; PK-PD: Pharmacokinetic - Pharmacodynamic; Q: Effluent flow rate; S: Sieving coefficient; t_1/2: _Half life; TBC: Total Body Clearance; t_inf: _Infusion duration time; V_d: _Volume of distribution; V_dss: _Volume of distribution at steady state; λ_z: _First order elimination rate constant; τ: Dosage interval.

## Competing interests

The authors declare that they have no competing interests.

## Authors' contributions

OC, CG, MD and AS initiated and supervised the study from its inception. CD devised study protocols and completed clinical trial applications. AS and CD co-ordinated sample acquisition. AS undertook sample analysis under the supervision of OC. AS and DD completed the pharmacokinetic analysis and modelling. AS and OC authored the first draft, with review and additions by DD, MD and CG. AS, OC, CG, MD, CD and DD read and approved the final manuscript.

## Pre-publication history

The pre-publication history for this paper can be accessed here:

http://www.biomedcentral.com/1472-6904/11/11/prepub
